# Complete Rupture of Testis After Blunt Trauma

**DOI:** 10.7759/cureus.47968

**Published:** 2023-10-30

**Authors:** Muhammad Shams, Yousaf Tanveer, Maryam Iqbal

**Affiliations:** 1 Urology, Royal Bornemouth Hospital, Bournemouth, GBR; 2 General Surgery, Cavan General Hospital, Cavan, IRL; 3 Internal Medicine, Sheikh Zayed Medical College and Hospital, Rahim Yar Khan, PAK

**Keywords:** scrotal pain, scrotal trauma, ultrasound scrotum, orchidectomy, testes

## Abstract

Testicular trauma can be classified aetiologically as blunt or penetrative. Bicycles and motorbikes are considered high risk for road traffic accidents accounting for 9-17% % of all blunt trauma. We present a case of assessment and management of isolated blunt testicular trauma in a tertiary care hospital.

A 36-year-old gentleman presented to the accident and emergency department with testicular pain after a road traffic accident while riding a motorcycle. On arrival, he was fully conscious and denied any other injuries. On examination, he had bruising and gross swelling of the right hemiscrotum. Ultrasound of the scrotum revealed testicular rupture and emergency exploration was undertaken which confirmed the diagnosis. Testis was non-salvageable therefore orchidectomy was done. The patient had unremarkable post-operative recovery.

A majority of testicular ruptures are secondary to blunt trauma mainly caused by sport-related injuries and road traffic accidents. Ultrasonography remains a non-invasive modality to investigate testicular injuries with a sensitivity of 100%. When not available in an emergency setting, scrotal exploration should be undertaken for both diagnostic and therapeutic purposes. Surgical repair done within 72 hours yields a 90% salvage rate after which the salvage rate is reduced significantly. Tumor markers should be checked in patients managed conservatively.

Early assessment and diagnosis are crucial in the management of acute testicular rupture. Early intervention can salvage injured testes and an orchidectomy can be avoided.

## Introduction

Testicular trauma is a rare occurrence. However, forceful injuries to the scrotum can cause relatively uncommon complications which should be considered while evaluating the patients presenting with such injuries. Testicular trauma can be classified aetiologically as blunt or penetrative. However blunt trauma accounts for a majority of cases mainly caused by sporting injuries, interpersonal violence, and road traffic accidents affecting males aged 15-40 years [1]. Although incidences of testicular rupture secondary to blunt trauma have been reported in the past, the completely isolated rupture of the testis is rare. We present a case of assessment and management of isolated blunt testicular trauma in a tertiary care hospital.

## Case presentation

A 36-year-old male came to the accident and emergency department of a tertiary care hospital with scrotal pain following a road traffic accident. He was riding a motorcycle when he fell off after a collision landing on the tank of the bike. He immediately developed unilateral right scrotal pain. Subsequently, he developed nausea but there was no abdominal pain or injury to any other part of the body. On arrival at the accident and emergency department, he was fully conscious. He was fit and well with no past medical history and did not take any regular medications.

On examination, his Glasgow Coma Scale (GCS) was 15 with unremarkable vitals. On examination, his abdomen was soft and non-tender. On examination of the external genitalia, he was found to have bruising of the right hemiscrotum. The right side was swollen grossly and tender on examination with normal left hemiscrotum. He did not have any other injuries.

**Figure 1:**
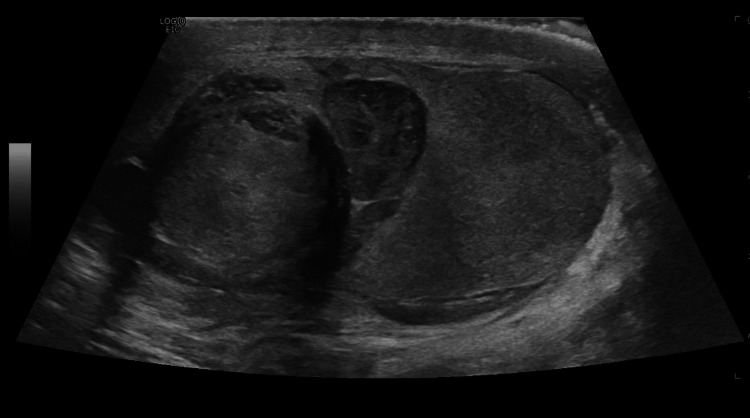
Ultrasound of the testis demonstrating rupture of testis with associated hematoma

Blood tests were unremarkable and an urgent ultrasound scan of the scrotum was arranged by the urology team. It revealed a disrupted contour with a 35mm heterogeneous area in the superior aspect of the right testis suggestive of hematoma and testicular rupture. No flow was demonstrated within the remainder of the testis with color doppler. The left testis and epididymis were normal. Figure 1 below shows the ultrasound of the testis which showed rupture of the testis and associated hematoma. 

Emergency scrotal exploration was undertaken under general anesthesia within two hours of presentation. A midline raphe incision was made and the right testis was delivered. About 60% of the testis was completely separate from the total split with surrounding blood clots. Residual tissue was purple and dead with only a tiny volume of the testis with decent color. Epididymis appeared normal. A decision was made to proceed with orchidectomy as the testis was non-salvageable. Good hemostasis was achieved by removing the clots from the scrotal pouch and use of monopolar diathermy. A corrugated drain was left in situ.

The patient recovered well and the drain was removed the next day after review. He was discharged one-day post-operation and was followed up in the outpatient department with testosterone levels in three months.

## Discussion

A majority of testicular ruptures diagnosed are secondary to blunt trauma mainly caused by sport-related injuries [2]. The second most common cause is road traffic accidents involving motor vehicles and motorbikes accounting for between 9% to 17% of injuries. The rest of the causes include falls and straddle injuries. A detailed history and examination according to advanced trauma life support (ATLS) guidelines should be undertaken to reach an appropriate diagnosis. Ultrasonography is the non-invasive modality of choice for investigating suspected testicular trauma and if not available, scrotal exploration is considered a diagnostic and therapeutic approach to such suspected injuries. Compared to surgical findings, the sensitivity of ultrasound scans in testicular trauma is 100% with a specificity of 93.5%.

The testis is covered by a tough fibrous covering of tunica albuginea and disruption of this layer or bulging of seminiferous tubules defines testicular rupture. It happens when the testis is forcefully hit against the pubis as a result of sudden deceleration and studies show that a force of 50kg is sufficient for testicular rupture to occur [3,4]. Scrotal exploration is usually necessary to determine the extent of the injury, washout of the wound, and control hemorrhage. If the tunica albuginea is breached, debridement of dead seminiferous tubules and primary closure of the tunica albuginea should be performed [5]. Surgical repair done within 72 hours will yield a 90% salvage rate after which the salvage rate is diminished to 45%. Emergency orchidectomy is rarely indicated unless the testis is non-salvageable and is completely shattered or infarcted. Serum tumor marker levels such as alpha-fetoprotein (AFP), beta human chorionic gonadotropin (hCG), and lactate dehydrogenase (LDH) should be checked in patients who are managed conservatively. Assuming adequate function of the contralateral testis, unilateral removal of the testis should not compromise the sexual function and fertility. In such patients, follow-up with testosterone levels can be offered.

## Conclusions

Early assessment and diagnosis are important in the management of testicular trauma. Ultrasonography is an excellent modality to diagnose the extent of testicular trauma and can guide in the timely management of such injuries. Exploration of the scrotum should be done when in doubt and should be considered the first line of management in all penetrating injuries.
